# Modeling ^18^F-FDG Kinetics during Acute Lung Injury: Experimental Data and Estimation Errors

**DOI:** 10.1371/journal.pone.0047588

**Published:** 2012-10-31

**Authors:** A. Susanne Dittrich, Tilo Winkler, Tyler Wellman, Nicolas de Prost, Guido Musch, R. Scott Harris, Marcos F. Vidal Melo

**Affiliations:** 1 Department of Anesthesia, Critical Care and Pain Medicine, Massachusetts General Hospital and Harvard Medical School, Boston, Massachusetts, United States of America; 2 Department of Medicine (Pulmonary and Critical Care Unit), Massachusetts General Hospital and Harvard Medical School, Boston, Massachusetts, United States of America; 3 Department of Anesthesia and Intensive Care Therapy, University Hospital Dresden, Dresden, Germany; D'or Institute of Research and Education, Brazil

## Abstract

**Background:**

There is increasing interest in Positron Emission Tomography (PET) of 2-deoxy-2-[18F]flouro-D-glucose (^18^F-FDG) to evaluate pulmonary inflammation during acute lung injury (ALI). We assessed the effect of extra-vascular lung water on estimates of ^18^F-FDG-kinetics parameters in experimental and simulated data using the Patlak and Sokoloff methods, and our recently proposed four-compartment model.

**Methodology/Principal Findings:**

Eleven sheep underwent unilateral lung lavage and 4 h mechanical ventilation. Five sheep received intravenous endotoxin (10 ng/kg/min). Dynamic ^18^F-FDG PET was performed at the end of the 4 h period. ^18^F-FDG net uptake rate (Ki), phosphorylation rate (k_3_), and volume of distribution (F_e_) were estimated in three isogravitational regions for each method. Simulations of normal and ALI ^18^F-FDG-kinetics were conducted to study the dependence of estimated parameters on the transport rate constants to (k_5_) and from (k_6_) the extra-vascular extra-cellular compartment. The four-compartment model described 85.7% of the studied ^18^F-FDG-kinetics better than the Sokoloff model. Relative to the four-compartment model the Sokoloff model exhibited a consistent positive bias in Ki (3.32 [1.30–5.65] 10^−4^/min, p<0.001) and showed inaccurate estimates of the parameters composing Ki (k_3_ and F_e_), even when Ki was similar for those methods. In simulations, errors in estimates of Ki due to the extra-vascular extra-cellular compartment depended on both k_5_ and k_5_/k_6_, with errors for the Patlak and Sokoloff methods of 0.02 [−0.01–0.18] and 0.40 [0.18–0.60] 10^−3^/min for normal lungs and of −0.47 [−0.89–0.72] and 2.35 [0.85–3.68] 10^−3^/min in ALI.

**Conclusions/Significance:**

^18^F-FDG accumulation in lung extra-vascular fluid, which is commonly increased during lung injury, can result in substantial estimation errors using the traditional Patlak and Sokoloff methods. These errors depend on the extra-vascular extra-cellular compartment volume and its transport rates with other compartments. The four-compartment model provides more accurate quantification of ^18^F-FDG-kinetics than those methods in the presence of increased extra-vascular fluid.

## Introduction

Acute lung injury (ALI) and the acute respiratory distress syndrome (ARDS) are inflammatory conditions that cause significant morbidity and mortality in critically ill patients [1], [2]. Noninvasive measurement of the magnitude and spatial distribution of inflammation in the lungs could be valuable to better understand those conditions, evaluate treatment response, and manage patients. For this reason, there is increasing interest in positron emission tomography (PET) imaging using 2-deoxy-2-[^18^F]fluoro-D-glucose (^18^F-FDG) to study regional inflammation in ALI/ARDS [3]–[6].

^18^F-FDG-PET is based on the principle that ^18^F-FDG is taken up by cells through the same pathways as glucose, then phosphorylated and trapped in the cells such that the intracellular ^18^F-FDG concentration increases in proportion to the cells glucose utilization rate. Thus, in the acutely inflamed non-tumoral lung, intravenous ^18^F-FDG is predominantly taken up by the most metabolically active inflammatory cells [7] with an important contribution from neutrophils [4], [8]–[11]. The ^18^F-FDG signal has accordingly been proposed as a method to quantify the activity and number of neutrophils [4], [7], [8], [10], [12]. Since neutrophils are key modulators of the magnitude of injury during ALI/ARDS [13], [14], pulmonary ^18^F-FDG imaging could be a valuable tool to noninvasively investigate regional inflammation in those conditions.

However, characteristics of ^18^F^-^FDG distribution in lung tissue could produce estimation errors in ^18^F-FDG kinetics. Methods to quantify ^18^F-FDG kinetics, the Patlak approach [15] and Sokoloff's three-compartment model [16], have been developed and applied to the study of solid organs such as the brain [15], [16], the heart [17] and the liver [18]. In contrast to those solid organs, the lung has significantly lower basal glucose consumption [19] and larger edema/tissue ratio in cases of organ injury [20]. In particular, an increase in lung water, a common finding during ALI/ARDS, would increase the volume of distribution for ^18^F-FDG, with accumulation of ^18^F-FDG in lung tissue independent of lung inflammation. This problem could substantially influence the ability of ^18^F-FDG kinetics parameters to accurately quantify inflammatory processes.

We recently showed that a lung-specific four-compartment model including a compartment specifically conceptualized to represent the extra-vascular extra-cellular space provided a better fit to the ^18^F-FDG kinetics than the Sokoloff model during smoke inhalation and ventilator-induced lung injury [12]. This improvement was attributed to more accurate modeling of ^18^F-FDG accumulation in edematous and flooded lung tissue. However, it is not known how this additional volume of distribution quantitatively affects the net ^18^F-FDG uptake rate (Ki) or its components related to the rate of ^18^F-FDG phosphorylation (k_3_) and tissue volume of distribution (F_e_). These parameters are especially important in the setting of ALI since they may provide information on neutrophil numbers and activity [12].

We hypothesized that the presence of an additional ^18^F-FDG distribution volume in the form of lung edema or alveolar flooding will lead to systematic errors in ^18^F-FDG kinetics parameters estimated with the Patlak and Sokoloff methods. Due to accumulation of ^18^F-FDG in this volume, we expect that net ^18^F-FDG uptake rates will be overestimated by those methods. Such errors should be reduced by using the four-compartment model, which accounts for such a distribution volume. Based on these hypotheses, our aims were to

1. Compare the parameters associated with volumes of distribution and ^18^F-FDG uptake estimated by the Patlak, Sokoloff, and four-compartment methods under different types and severities of regional lung injury;
2. Identify the causes for differences in parameter estimates among the methods; and
3. Quantify the effect of the presence of an extra-vascular extra-cellular compartment on parameter estimates provided by Patlak and Sokoloff methods, using theoretical simulations.

## Materials and Methods

### Experimental Preparation

The experimental procedures were approved by the Subcommittee on Research Animal Care (SRAC), which serves as the Institutional Animal Care and Use Committee (IACUC) for the Massachusetts General Hospital (Protocol Number: 2006N000129). All surgery was performed under general intravenous anesthesia, and all efforts were made to minimize suffering. In order to investigate the effect of lung water content in the regional quantification of pulmonary ^18^F-FDG kinetics, we used sheep models of continuous systemic endotoxemia and of unilateral surfactant depletion with alveolar saline lavage and moderately aggressive mechanical ventilation.

Eleven sheep (21.4±1.5 kg) were anesthetized, intubated and mechanically ventilated. A femoral artery was percutaneously cannulated for arterial blood samples and blood pressure monitoring. A 9F introducer and a pulmonary artery catheter were inserted using the right internal jugular vein. A tracheotomy was performed and a 35-French left-sided double-lumen endobronchial tube was inserted. After increasing the FiO_2_ to 1, left lung surfactant depletion was produced with alveolar saline lavage to a PaO_2_/FiO_2_ ≤200 mmHg. Starting from the supine position, aliquots of ∼400 mL were instilled in the airways up to a pressure level of ∼30 cmH_2_O. After three aliquots, the animal was turned prone for further aliquots, to homogenize lavage of ventral and dorsal regions. A median volume of 1900 mL (1800–2600) was necessary to reach the PaO_2_/FiO_2_ target. An average of 400 mL (300–600) remained in the lungs, which is consistent with previous studies [21]. The double-lumen endobronchial tube was then replaced by a regular endotracheal tube and double lung ventilation was resumed.

### Experimental Protocol

The animals were placed supine in the PET scanner (Scanditronix PC4096; General Electric, Milwaukee, WI) with the dome of the diaphragm just outside the field of view. Mechanical ventilation was applied for four hours with the following settings: PEEP = 10 cmH_2_O, FiO_2_ = 0.6, I∶E ratio 1∶2, tidal volume adjusted to a plateau pressure of 30 cmH_2_O and respiratory rate adjusted to normocapnia. PET scans performed at baseline and at the end of the four-hour period included a transmission scan and an emission scan following an intravenous ^13^NN-saline bolus infusion [22]. ^18^F-FDG PET scans were acquired only after the final set of ^13^NN scans. After the baseline scans, five sheep received a continuous 10 ng/kg/min intravenous infusion of Escherichia coli endotoxin (lipopolysaccharide, *LPS,* O55:B5, List Biological Laboratories Inc, California) while six did not.

In this manner, four regional pulmonary pathophysiological conditions were studied:

1. healthy lung, i.e., no LPS and no lung lavage (LPS−, Lav−)
2. lung exposed to bronchoalveolar lavage but not to endotoxin (LPS−, Lav+)
3. lung exposed to systemic endotoxin but not to lavage (LPS+, Lav−)
4. lung exposed to systemic endotoxin and bronchoalveolar lavage (LPS+, Lav+)

Following the ^18^F-FDG imaging, animals were euthanized and lungs were harvested. Blocks of lung tissue (∼1 cm^3^) were sampled from ventral, middle and dorsal regions of each lung before fixation. They were weighed, dried for 4 days at 80°C and weighed again. The wet-to-dry ratio was calculated as the ratio of the weight measured shortly after the lung extraction and the weight measured after the drying period.

### PET Imaging Protocol and Processing

PET imaging methods and analysis have been previously described in detail [4], [22]–[24]. Briefly, the PET camera acquired 15 transverse cross-sectional slices of 6.5-mm thickness providing 3-dimensional information over a 9.7-cm-long field of view corresponding to ∼70% of the total lung volume. Resulting images consisted of an interpolated matrix of 128×128×15 voxels. Three different types of PET scans were performed:

1. Transmission scans were obtained over 10 min prior to each emission scan to correct for attenuation in emission scans and to calculate regional gas (F_gas_) and tissue fraction (F_tissue_ = 1-F_gas_-F_B_), where F_B_ is the fractional volume of blood derived from ^18^F-FDG kinetics using the four-compartment model [12].
2. ^13^NN emission scans starting simultaneously with a bolus injection of ^13^NN-saline during a 60 s apnea at mean lung volume were used to obtain images of the perfused lung tissue for delineation of the lung field [22], [24], [25].
3. ^18^F-FDG emission scans were obtained for quantification of regional ^18^F-FDG kinetics. After ^13^NN clearance, ^18^F-FDG (5–10 mCi) was infused at a constant rate through the jugular catheter over 60 s and, simultaneous with the beginning of ^18^F-FDG infusion, sequential PET frames (6×30 s, 7×60 s, 15×120 s, 1×300 s, 3×600 s) were acquired over 75 min while plasma samples were collected from pulmonary arterial blood at time points: 5.5, 9.5, 25, 37, and 42.5 min. ^18^F-FDG PET scans were acquired only after injury because of the 110-min half-life of ^18^F-FDG.

Lung masks were created by combining aerated lung regions from transmission scans with perfused regions from ^13^NN emissions scans. The lung field was divided into three equispaced regions of interest (ROIs) along the gravitational axis (non-dependent, middle and dependent), which were used to quantify regional tissue fraction and ^18^F-FDG kinetics.

### Modeling of ^18^F-FDG Kinetics

The net uptake rate of ^18^F-FDG in lung parenchyma (Ki), as well as volumes of tracer distribution in lung tissue, were computed in each ROI using the three methods described below [26]. For each animal, an image-derived input function of ^18^F-FDG in pulmonary arterial plasma was computed as previously described [12] and used for all models and ROIs.

### Patlak Method

The Patlak graphical method consisted of plotting the ^18^F-FDG activity in a ROI normalized to plasma activity against the integral of plasma activity normalized to plasma activity [15]. The net ^18^F-FDG uptake rate (Ki_P_) was calculated from the slope of the linear regression using a time window from 15 minutes to the end of the imaging protocol. The ordinate intercept of the regression line at time = 0 (Y-intercept) gave a measure of the distribution volume of ^18^F-FDG [15].

### Sokoloff Model

Sokoloff's three-compartment model encompasses a blood and two tissue compartments corresponding to a precursor tissue compartment and a metabolized phosphorylated ^18^F-FDG compartment [16]. In this model, k_1_ is the transfer rate of tracer from plasma into a precursor compartment for ^18^F-FDG phosphorylation, k_2_ is the transfer rate of tracer from the precursor compartment back into the blood, and k_3_ is the rate constant describing the phosphorylation of ^18^F-FDG to ^18^F-FDG-6-phosphate, a process assumed to be proportional to hexokinase activity [16]. From these, the net uptake rate of ^18^F-FDG (Ki_S_) and the fractional distribution volume of the extra-vascular precursor compartment (F_e_) were computed:
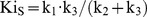
(1)

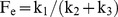
(2)Combining equations 1 and 2,
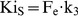
(3)The model was fitted to the ^18^F-FDG kinetics using the multi-level coordinate search (MCS) method to find the parameter set minimizing the mean squared error of the model fit [24], [27].

### Four-Compartment Model

**Figure 1:**
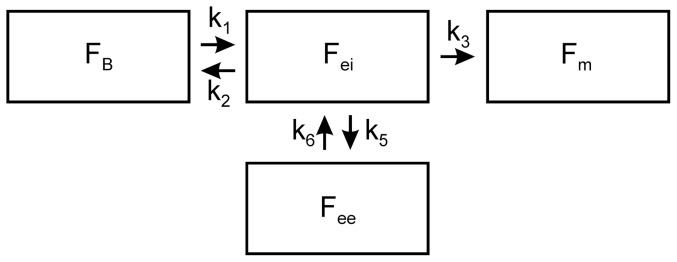
Four-Compartment Model. Schematic of the lung-specific four-compartment model of ^18^F-FDG kinetics including an extra-vascular extra-cellular compartment to account for ^18^F-FDG that is not directly available for phosphorylation, such as ^18^F-FDG in lung water. Note the functional distinction between extra-vascular extra-cellular compartment, which is a non-substrate compartment so that ^18^F-FDG is not available for phosphorylation, and the precursor compartment where ^18^F-FDG is available for phosphorylation.

The four-compartment model described by Schroeder et al. [12] aims at describing the ^18^F-FDG kinetics in lungs with ALI by addition of an extra-vascular extra-cellular tracer distribution volume exchanging tracer with the extra-vascular intra-cellular precursor compartment. The important functional distinction between the precursor compartment and the extra-vascular extra-cellular compartment is a non-substrate compartment, in which ^18^F-FDG is not available for phosphorylation by hexokinase while ^18^F-FDG in the precursor pool is available for phosphorylation (Fig. 1). Accordingly, the model includes the constants k_5_ and k_6_ to represent the forward and backward transfer rates of ^18^F-FDG between the precursor and the extra-vascular extra-cellular compartment, in addition to rate constants k_1_, k_2_, and k_3_ from the Sokoloff model. All rate constants, as well as the fractional blood volume (F_B_), were estimated using the same MCS method as for the Sokoloff model [24], [27]. The tracer in a region of interest was thus partitioned in distribution volumes conceptualized as: (a) the pulmonary blood plasma; (b) the extra-vascular intra-cellular precursor compartment (F_ei_); (c) the metabolized, trapped tracer compartment; and (d) the extra-vascular extra-cellular compartment (F_ee_). F_ei_ was computed as in Equation 2, while F_ee_ and the net uptake rate Ki_F_ were computed as [12]:
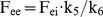
(4)

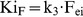
(5)Note that the ratio between the distribution volumes F_ee_ and F_ei_ is given by the ratio of the transfer rates k_5_/k_6_.

### Simulations of ^18^F-FDG Kinetics

Computational simulations were used to generate ^18^F-FDG kinetics for different levels of lung edema in order to study the effect of lung edema/flooding on the parameters of the different ^18^F-FDG kinetics models. This theoretical approach was used to analyze the effect of an extra-vascular extra-cellular volume of distribution (F_ee_) on the error in parameter estimation. The use of simulations allowed us to assess the performance of the different models systematically and under ideal conditions, without measurement errors involved in experimental data. Specifically, we focused on errors in k_3_, F_e_, and Ki_S_ from the Sokoloff model and Ki_P_ from the Patlak method because the net uptake rates (Kip and Kis) have been frequently used in animal and clinical experiments as a measure of cellular metabolic activation and neutrophilic inflammation during acute lung injury [3]–[6] and k_3_ and F_e_ are the variables that determine the net uptake rate (Eq. 3). In order to study different tissue conditions that are pathophysiologically relevant, we derived sets of model parameters from one normal non-dependent (LPS−, Lav−) and one injured dependent (LPS+, Lav+) experimental lung region (Table 1). Based on these datasets, ^18^F-FDG kinetics of one lung region with low (LPS−, Lav−) and one with high ^18^F-FDG net uptake rates (LPS+, Lav+) were simulated (Table 1). The influence of the extra-vascular extra-cellular compartment was explored by systematically varying two model parameters:

1. the ratio of k_5_/k_6_, which expresses the ratio of the volume of the extra-vascular extra-cellular compartment to the volume of the extra-vascular intra-cellular compartment; and
2. the absolute value of k_5_, which represents the rate constant from the extra-vascular intra-cellular precursor compartment into the extra-vascular extra-cellular compartment.

**Table 1:** Estimates of the Patlak, Sokoloff and Four-Compartment Method for two representative Lung Regions of Interest in a healthy Lung (LPS−, Lav−) and a Lung exposed to bronchoalveolar Lavage and systemic Endotoxin (LPS+, Lav+).

Simulation	LPS−, Lav−	LPS+, Lav+
ROI	Non-Dependent	Dependent
*Patlak Method*		
**Ki_P_ (10^−3^/min)**	0.71	15.80
**Y-intercept**	0.13	0.85
*Sokoloff Model*		
**F_B_**	0.07	0.09
**k_1_ (10^−1^/min)**	0.13	0.88
**k_2_ (1/min)**	0.25	0.12
**k_1_/k_2_**	0.05	0.71
**k_3_ (10^−2^/min)**	1.77	3.08
**Ki_S_ (10^−3^/min)**	0.86	17.51
**F_e_**	0.05	0.57
*Four-Compartment Model*		
**F_B_**	0.04	0.07
**k_1_ (10^−1^/min)**	1.00	1.13
**k_2_ (1/min)**	1.94	0.23
**k_1_/k_2_**	0.05	0.48
**k_3_ (10^−2^/min)**	1.43	3.68
**Ki_F_ (10^−3^/min)**	0.73	15.31
**F_ei_**	0.05	0.42
**k_5_ (1/min)**	0.07	0.04
**k_6_ (1/min)**	0.11	0.06
**k_5_/k_6_**	0.62	0.75
**F_ee_**	0.03	0.31

The k_5_ and k_5_/k_6_ values ranging from zero to the 75^th^ percentile of the k_5_ and the k_5_/k_6_ distributions obtained from the experimental data were combined with k_1_, k_2_ and k_3_ taken from estimates of the four-compartment model for the ROIs shown in Table 1. Those k_5_ and k_5_/k_6_ ranges were divided in nine steps.

Tracer kinetics were generated for each set of parameters by using Euler's method for numerical solution of the model's differential equations. Initial activity in all compartments was set to zero, and plasma input functions were taken from the experimental data. From the simulated tracer kinetics, parameters for the Sokoloff and Patlak method were estimated as described above for imaged kinetics. In the lowest simulated ranges of k_5_/k_6_, the value of k_6_ became unreasonably high for Euler's method, and the tracer content of the extra-vascular extra-cellular compartment became negligible. Thus, we excluded the simulations with the lowest k_5_ and k_5_/k_6_ values from further analysis and values from the 75th percentile/9 to the 75th percentile of distributions obtained from the experimental data were studied. The errors in the parameters estimated from the simulated time-activity curves were quantified as the absolute paired difference of the estimated (Ki_P_, Ki_S_, k_3S_, k_1S_, k_2S_, F_e_) and the original parameters used for the simulation (Ki_F_, k_1F_, k_2F,_ k_3F_ and F_ei_), i.e.:
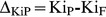
(6)

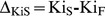
(7)

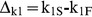
(8)

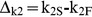
(9)

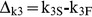
(10)

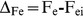
(11)and as the paired differences normalized by the original parameter used for the simulation Ki_F_, k_3F_ and F_ei_:
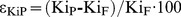
(12)

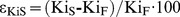
(13)

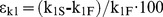
(14)

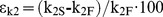
(15)

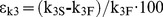
(16)

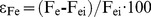
(17)where Δ_KiP_ is the absolute error in Ki_P_, Δ_KiS_ in Ki_S_, Δ_k1_ in k_1S_, Δ_k2_ in k_2S_, Δ_k3_ in k_3S_ and Δ_Fe_ in F_e_. The relative errors are ε_KiP_ for Ki_P_, ε_KiS_ for Ki_S_, ε_k1_ for k_1S_, ε_k2_ for k_2S_, ε_k3_ for k_3S_ and ε_Fe_ for F_e_.

To explore the causes of errors in k_3_ and F_e_ for the Sokoloff model, we examined how the estimated tracer distribution within the Sokoloff model changes to account for the additional tracer contained in the extra-vascular extra-cellular compartment. In order to quantify this change in the estimated tracer distribution, for each of the substrate and metabolized compartments, we computed the integral of the compartment activity according to the Sokoloff model over the imaging duration, divided by the integral of the activity of that compartment in the simulated four-compartment kinetics. Ratios greater than one indicate overestimation of tracer activity in that compartment due to the presence of tracer in the extra-vascular extra-cellular compartment. These ratios were computed over the full range of k_5_ for two different values of k_5_/k_6_ (0.3 and 1.1) in the “LPS+, Lav+” animal.

### Statistical Analysis

Variables were tested for normality using Shapiro-Wilk test. Normally distributed data were expressed as mean ± standard deviation, and as median [interquartile range 25–75%] otherwise.

To evaluate the relationship of the estimated volumes of distribution and the experimental wet-to-dry ratio, Spearman rank correlation was used.

Bland-Altman plots were constructed to compare Ki_P_ of the Patlak and k_3_, F_e_ and Ki_S_ of the Sokoloff method with the corresponding parameters of the four-compartment model. To determine whether these estimates were under- or overestimated in dependency of their absolute value, Spearman rank correlation was applied. Moreover, Spearman's rank correlation was used to analyze the interaction of the biases in k_1_, k_2_, k_1_/k_2_ and F_e_ estimated with the Sokoloff model. Wilcoxon rank sum test was used to compare the paired differences Ki_P_-Ki_F_ and Ki_S_-Ki_F_ with zero.

Outliers of extremely high k_5_/k_3_ ratios occurred in some cases when the extra-vascular extra-cellular compartment slowly accumulated activity over the imaging period, similar to the trapping of activity in the metabolized compartment, such that the parameter estimation technique was unable to distinguish between the extra-vascular extra-cellular compartment and the metabolized compartment. The Hampel identifier was used to detect and exclude outliers of k_5_/k_3_ ratios with |k_5_/k_3i_ - median|>5.0 * (median absolute deviation) [28]. Three ROIs were excluded based on this criterion, resulting in the final sample sizes for each group of: LPS−, Lav−, n = 18; LPS−, Lav+, n = 17; LPS+, Lav−, n = 13; and LPS+, Lav+, n = 15.

The Akaike information criterion (AIC) [29] was used to quantify the goodness of fit to the ^18^F-FDG kinetics of the Sokoloff model (AIC_S_) and the four-compartment model (AIC_F_).

Experimental wet-to-dry ratios and tissue fractions as well as parameters estimated from the “LPS−, Lav−” and the “LPS+, Lav+” simulations were compared using Wilcoxon's rank sum test. Parameters estimated by the Patlak method, the Sokoloff model and the four-compartment model were compared in the different studied conditions using Wilcoxon's signed-rank test. The level of significance was p<0.05. Multidimensional data were visualized as contour plots and four-dimensional contour plots [30].

## Results

### Experimental Data

Unilateral lavage increased wet-to-dry ratios in the lavaged lung for both LPS− (4.82 vs. 6.53, p<0.001) and LPS+ (6.95 vs. 9.41, p<0.05) conditions. This was accompanied by an increase in median density of the lavaged lungs as compared to the non-lavaged lungs (F_tissue_ = 0.36 vs. 0.24, p<0.001).

The four-compartment model provided a better description of regional tracer kinetics than the Sokoloff model in 54 of 63 studied isogravitational ROIs (85.7%). This was evidenced by the quantitative measure of goodness-of-fit AIC_F_, which was consistently lower than AIC_S_ (p<0.001, Table 2).

**Table 2:** Tracer Kinetics Parameters of the Patlak, the Sokoloff and the Four- Compartment Methods and experimental Wet-to-Dry Ratio.

	Median [interquartile range 25–75%]	Range	Correlation with Wet-to-Dry Ratio
*Patlak Method*			
**Ki_P_ (10^−3^/min)**	2.43 [1.49–4.68]	0.46–15.80	
**Y-intercept**	0.25 [0.17–0.40]	0.11–0.85	0.55***
*Sokoloff Model*			
**Ki_S_ (10^−3^/min)**	2.67 [1.79–5.21]	0.85–17.51	
**F_e_**	0.12 [0.09–0.21]	0.04–0.65	0.74***
*Four-Compartment Model*			
**Ki_F_ (10^−3^/min)**	2.28 [1.43–3.97]	0.50–15.31	
**F_ei_**	0.08 [0.07–0.14]	0.02–0.49	0.45***
**F_ee_**	0.08 [0.05–0.14]	0.00–0.67	0.62***
**F_ei_+F_ee_**	0.16 [0.11–0.32]	0.06–1.03	0.65***
**AIC_F_ - AIC_S_**	−27.84 [(−48.08)–(−12.53)]	−91.21–7.59	
*Experimental Wet-to-Dry Ratio (WD)*			
**WD**	6.25 [5.30–8.64]	4.29–17.78	

Values are shown as median [interquartile range 25–75%];

*** p<0.001.

**Figure 2:**
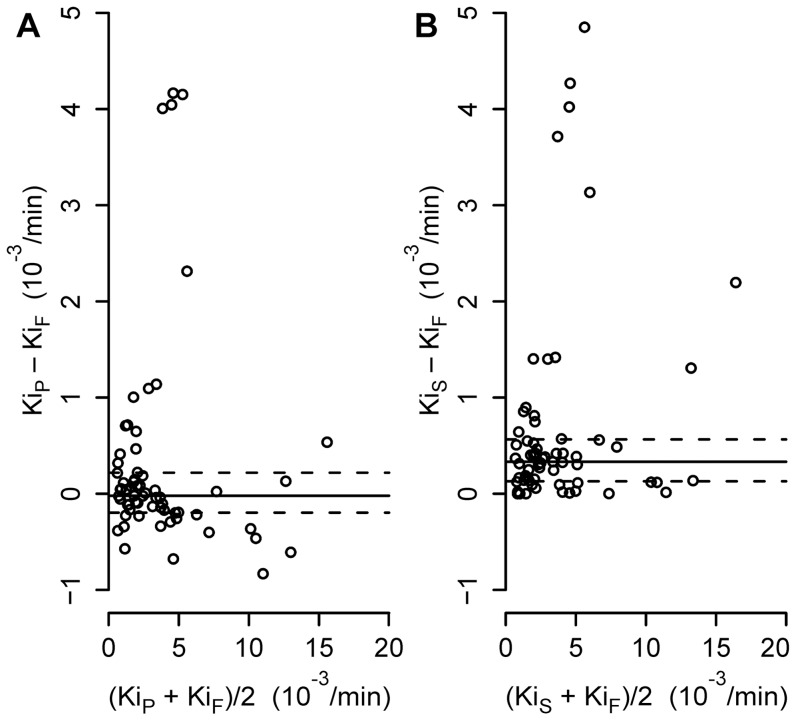
Bland-Altman Plots of Net Uptake Rates. Bland-Altman plots of (A) the net uptake rate Ki_P_ estimated by the Patlak graphical method (Ki_P_-Ki_F_ [10^−3^/min]) and (B) Ki_S_ estimated by the Sokoloff model versus Ki_F_ of the four-compartment model (Ki_S_-Ki_F_ [10^−3^/min]) in three isogravitational ROIs (non-dependent, middle, dependent), solid lines represent the median and dashed lines the interquartile range 25–75%.

Estimates of distribution volumes calculated by the four-compartment and the Sokoloff model and the Y-intercept of the Patlak method in the different studied conditions and ROIs provided a wide range of values, consistent with the wide range of experimental wet-to-dry ratios (Table 2). The estimated volumes of distribution were significantly correlated with wet-to-dry ratios (Table 2). The differences between net ^18^F-FDG uptake rates computed with the Patlak and four-compartment methods (Ki_P_-Ki_F_) were slightly negative and not significantly different from zero (−0.21 [−1.97–2.19] · 10^−4^/min, p = 0.87, Fig. 2A). In contrast, a Bland-Altman plot of the difference between Ki estimates of the Sokoloff and four-compartment models showed a bias (Ki_S_-Ki_F_), which was significantly greater than zero (3.32 [1.30–5.65] · 10^−4^/min, p<0.001, Fig. 2B), indicating the potential for overestimation of Ki using the Sokoloff method. Overall, the bias of Ki_S_-Ki_F_ was larger than that of Ki_P_-Ki_F_ (p<0.001).

**Figure 3:**
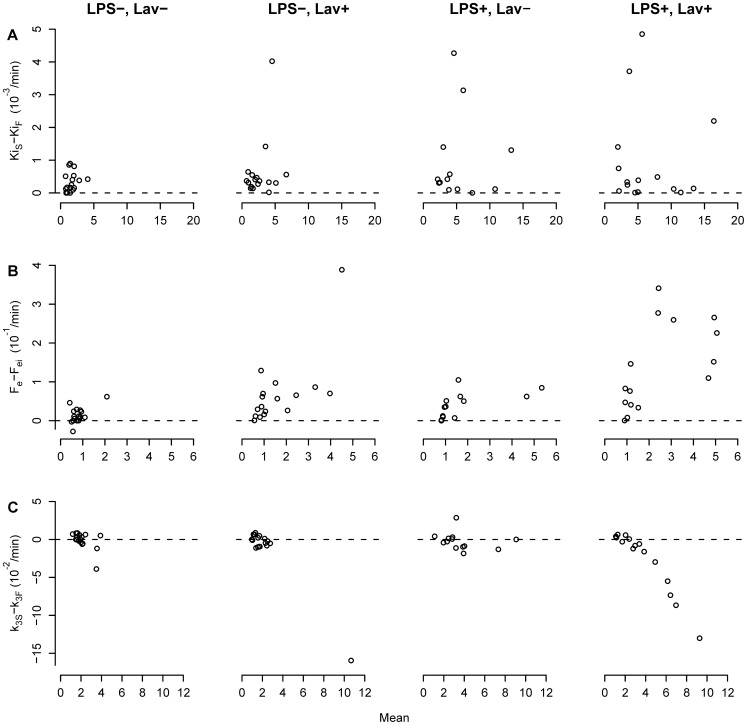
Bland-Altman Plots of k_3_ and F_e_. Bland-Altman plots comparing (A) the net ^18^F-FDG uptake rate Ki_S_ of the Sokoloff model with Ki_F_ of the four-compartment model, (B) the intracellular distribution volume F_e_ of the Sokoloff model with F_ei_ of the four-compartment model and (C) k_3S_ of the Sokoloff model with k_3F_ the four-compartment model of three isogravitational ROIs in healthy lungs (LPS−, Lav−), lungs exposed to bronchoalveolar lavage (LPS−, Lav+), lungs exposed to systemic endotoxin (LPS+, Lav−) and lungs exposed to systemic endotoxin and bronchoalveolar lavage (LPS+, Lav+). Note, that in the “LPS+, Lav+” condition the Sokoloff model tends to overestimate the fractional distribution volume of the precursor pool (F_e_) at high mean values of that volume and to underestimate the hexokinase activity (k_3S_) at high mean values of k_3_. In contrast, the net uptake rates Ki were similar for the four-compartment and Sokoloff models in high Ki ranges.

The Sokoloff model appeared to overestimate the extra-vascular volume of distribution F_e_ at higher mean values of that volume (r_s_ = 0.76, p<0.001, Fig. 3B) and to underestimate the phosphorylation rate k_3_ at higher mean values of k_3_ (r_s_ = −0.62, p<0.001, Fig. 3C). As a consequence, even when the overall ^18^F-FDG uptake rate was similar for the four-compartment and Sokoloff models (Fig. 3A), the corresponding values of its components k_3_ and F_e_ displayed a bias in the Sokoloff method as compared to the four-compartment model.

The differences between Sokoloff and four-compartment model estimates of transfer rates to (k_1S_-k_1F_) and from (k_2S_-k_2F_, k_3S_-k_3F_) the extra-vascular distribution volume F_e_ or F_ei_ showed a negative bias for the Sokoloff k_1_ (−3.27 [−5.15–−1.45] · 10^−2^/min), k_2_ (−4.30 [−7.55–−1.49] · 10^−1^/min) and k_3_ (−1.70 [−9.60–−3.69] · 10^−3^/min). The bias of the denominator composing F_e_ ( = k_1_/(k_2_+k_3_)) was substantially determined by the bias in k_2_, whose median was two orders of magnitude higher than the median bias in k_3_. The biases in k_1_ and k_2_ were correlated (r_s_ = 0.89, p<0.001), but the Sokoloff model led to a higher underestimation of k_2_ than k_1_ (p<0.001). Accordingly, the ratio k_1_/k_2_ exhibited a positive bias (4.76 [1.30–9.21] · 10^−2^/min) which was closely correlated with the bias in F_e_ (r_s_ = 0.99, p<0.001).

### Simulation Studies

**Figure 4:**
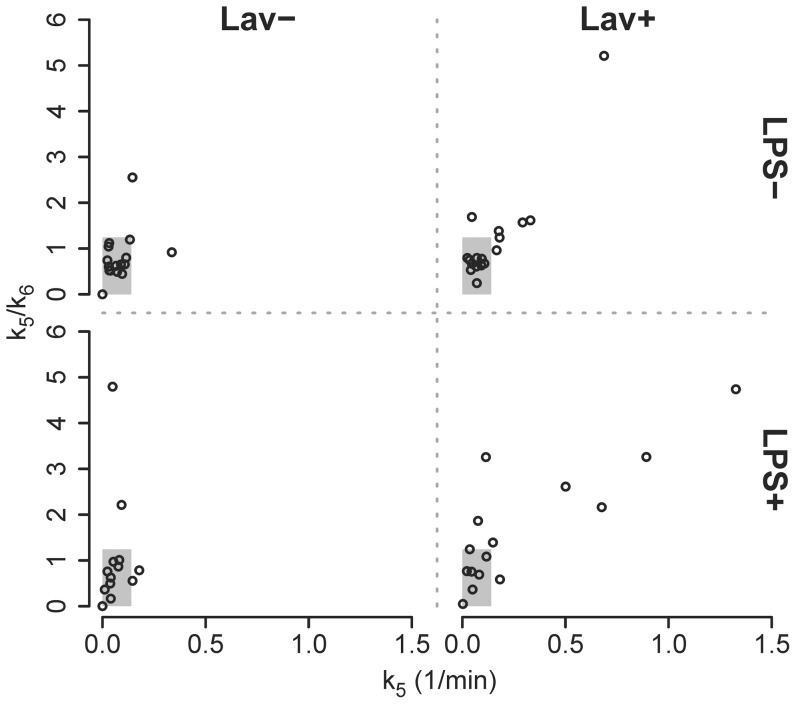
Parameters Defining the Extra-cellular Extra-vascular Compartment. Relationship between k_5_ and k_5_/k_6_ of three isogravitational ROIs in control lungs (LPS−, Lav−), lungs exposed to bronchoalveolar lavage (LPS−, Lav+), lungs exposed to systemic endotoxin (LPS+, Lav−) and lungs exposed to endotoxin and lavage (LPS+, Lav+). The highlighted gray area illustrates the range over which k_5_ and k_5_/k_6_ were varied in the simulations (0–75^th^ percentile of all experimental data).

Using the 75th percentile/9 and the 75th percentile of all experimental values of k_5_ and k_5_/k_6_ yielded parameter ranges for the simulation of 0.15 to 1.39 · 10^−1^/min and 0.14 to 1.24 (Fig. 4).

**Figure 5:**
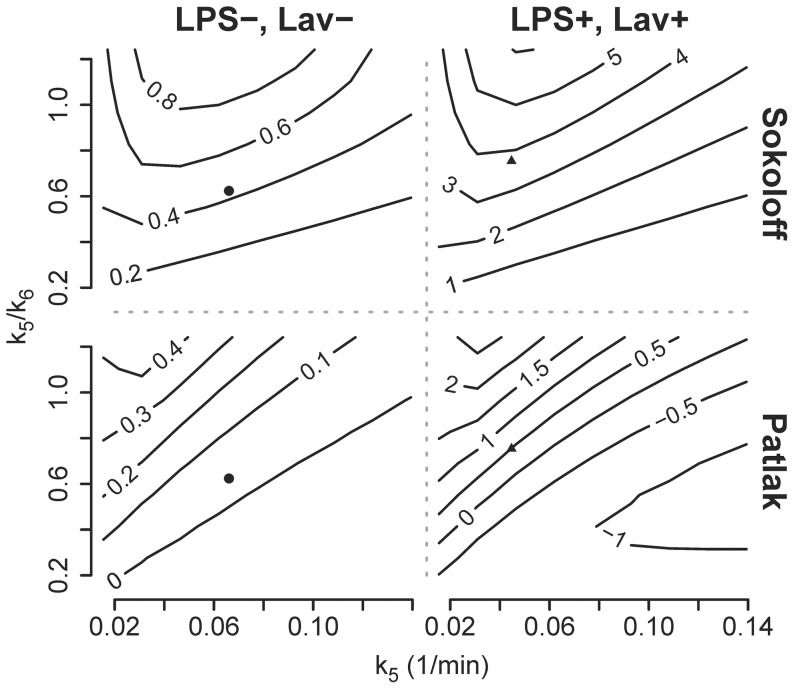
Errors in Net Uptake Rates Estimated by the Patlak and Sokoloff Methods. Contour plot showing the effect of k_5_ and k_5_/k_6_ on the absolute errors of Ki in the Sokoloff (Δ_KiS_ = Ki_S_-Ki_F_ [10^−3^/min]) and the Patlak methods (Δ_KiP_ = Ki_P_-Ki_F_ [10^−3^/min]) as compared to the four-compartment model (contour lines) in simulations of a healthy lung (LPS−, Lav−) and of a lung exposed to systemic endotoxin and bronchoalveolar lavage (LPS+, Lav+), original data points of the “LPS−, Lav−” simulation (•) and the “LPS+, Lav+” simulation (▴). Note that in both simulated conditions Δ_KiS_ is larger than Δ_KiP_. Both the Patlak method and the Sokoloff model show higher errors in the “LPS+, Lav+” simulation.

Simulations of the effects of pulmonary edema/flooding on Patlak and Sokoloff estimates of ^18^F-FDG kinetics parameters revealed that potential errors in those models' estimates of net uptake rate Ki depend on both the influx rate (k_5_) and the fractional volume (F_ee_ = F_ei_·k_5_/k_6_) of the extra-vascular extra-cellular compartment (Fig. 5, Supplementary Figure S1). Thus, errors in Ki_S_ and Ki_P_ theoretically depend on both the volume of edema fluid and the equilibration rate of that volume with ^18^F-FDG in the tissue. This finding was true for the two conditions tested: the normal lung (LPS−, Lav−) and the lung region injured with LPS and saline lavage (LPS+, Lav+, Table 1). For the Patlak method, the absolute error of the net uptake rate Ki_P_ followed a similar pattern in both simulated conditions (Table 3) with highest values in presence of small k_5_ and high k_5_/k_6_ values (Fig. 5). In the Sokoloff model, the absolute error of net uptake rate Δ_kiS_ also showed a similar behavior in the two studied conditions (Table 3) with maxima at small k_5_ and high k_5_/k_6_ (Fig. 5). In both simulations Δ_kiS_ reached values higher than Δ_kiP_ (Table 3). Thereby, in either model the absolute error of net uptake rate was higher in the “LPS+, Lav+” than in the “LPS−, Lav−” simulation. When corresponding relative errors ε_kiP_ and ε_kiS_ were computed, values were higher in the “LPS−, Lav−” than in the “LPS+, Lav+” simulation (Table 3).

**Table 3:** Absolute and relative Errors of estimated Parameters for the Patlak and the Sokoloff Model.

	LPS−, Lav−	LPS+, Lav+
*Patlak Method*		
**Δ_KiP_ (10^−3^/min)**	0.02 [−0.01–0.18]†††	−0.47 [−0.89–0.72]** ^,^ †††
**ε_KiP_ (%)**	2.7 [−1.3–24.0]†††	−3.1 [−5.8–4.7]*** ^,^ †††
*Sokoloff Model*		
**Δ_KiS_ (10^−3^/min)**	0.40 [0.18–0.60]	2.35 [0.85–3.68]***
**ε_KiS_ (%)**	55.2 [24.1–81.3]	15.3 [5.5–24.1]***
**Δ_k3_ (10^−2^/min)**	0.52 [0.18–1.05]	−0.43 [−0.82–0.03]***
**ε_k3_ (%)**	36.3 [12.7–73.2]	−11.8 [−22.2–0.8]***
**Δ_Fe_ (10^−1^/min)**	0.04 [0.01–0.07]	1.15 [0.55–1.86]***
**ε_Fe_ (%)**	6.9 [1.5–13.9]	27.7 [13.2–44.7]***

Values are shown as median [interquartile range 25–75%];

** p<0.01,

*** p<0.001 as compared to the “LPS−, Lav−” simulation;

††† p<0.001 as compared to the corresponding absolute respective relative error in Ki_S_.

**Figure 6:**
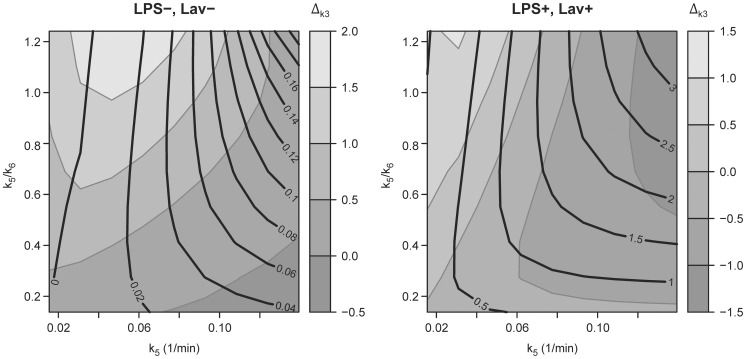
Errors in k_3_ and F_e_ Estimated by the Sokoloff Method. Errors in both k_3_ (Δ_k3S_ = k_3S_-k_3F_ [10^−2^/min]) as filled contours and F_e_ (Δ_Fe_ = F_e_-F_ei_ 10^−1^) as contour lines as function of k_5_/k_6_ and k_5_ in simulations of a healthy lung (LPS−, Lav−) and of a lung exposed to systemic endotoxin and bronchoalveolar lavage (LPS+, Lav+). Note that Δ_k3_ is higher in the simulation of the healthy lung and Δ_Fe_ is higher in the simulation of the injured lung.

Similarly to the observation in the experimental data, the Sokoloff model displayed errors in the individual components of the net uptake rate, e.g. the phosphorylation rate k_3S_ and the distribution volume of the extra-vascular precursor compartment F_e_ (Table 3 and Fig. 6). Absolute and relative errors of k_3S_ were higher in the “LPS−, Lav−” simulation than in the “LPS+, Lav+” simulation (Table 3 and Fig. 6). Accordingly, absolute and relative errors in F_e_ showed higher values in the “LPS+, Lav+” simulation than in the “LPS−, Lav−” simulation (Table 3 and Fig. 6). Δ_Fe_ and Δ_k3_ showed opposite trends in high k_5_ and k_5_/k_6_ ranges.

**Figure 7:**
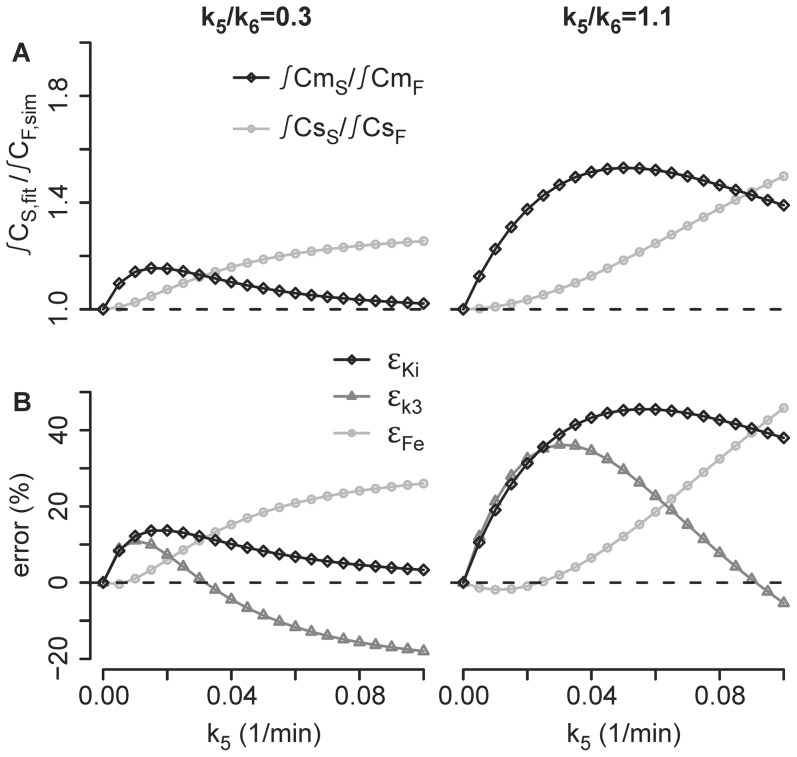
Compartment Activities and Estimation Errors as a Function of k_5_. (A) Integral of the compartment activity according to the Sokoloff model over the imaging duration (∫C_S,fit_), divided by the integral of the activity of that compartment in the simulation (∫C_F,sim_) for the substrate (∫Cs_S_/∫Cs_F_) and the metabolized compartments (∫Cm_S_/∫Cm_F_) versus k_5_ at a k_5_/k_6_ ratio of 0.3 and of 1.1; (B) relative errors of Ki (ε_KiS_ = (Ki_S_-Ki_F_)/Ki_F_·100), k_3_ (ε_k3S_ = (k_3S_-k_3F_)/k_3F_·100) and F_e_ (ε_Fe_ = (F_e_-F_ei_)/F_ei_·100) versus k_5_ at a k_5_/k_6_ ratio of 0.3 and of 1.1.

**Figure 8:**
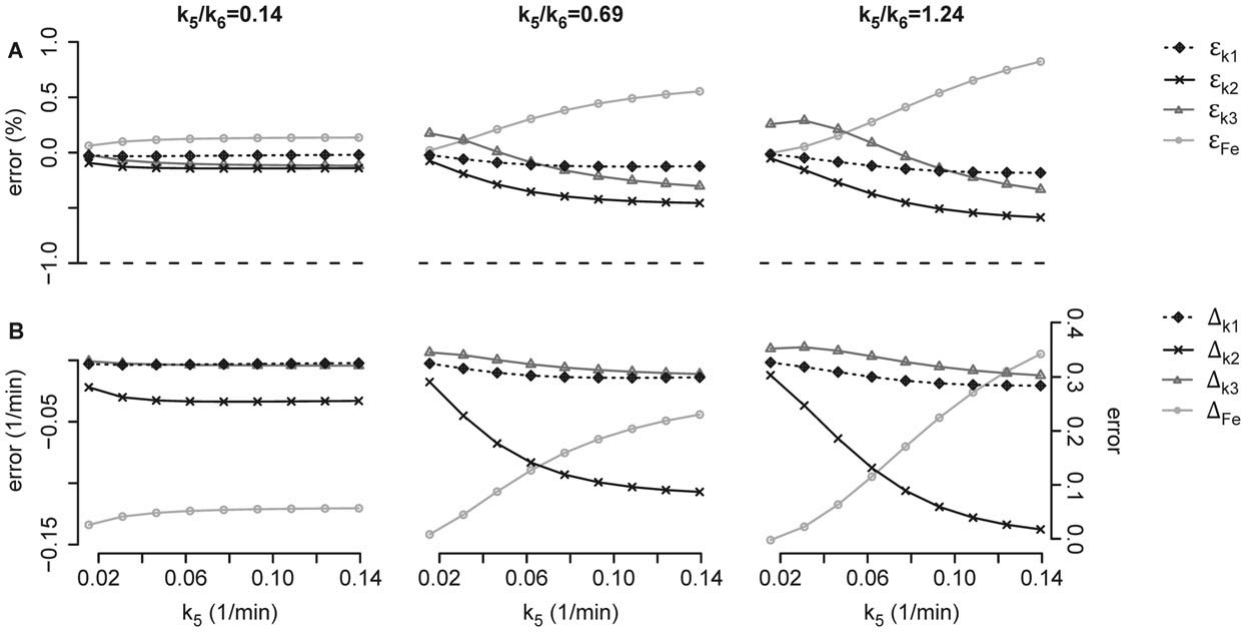
Estimation Errors as a Function of k_5_. (A) Relative errors of k_1_ (ε_k1_ = (k_1S_-k_1F_)/k_1F_ ·100), k_2_ (ε_k2_ = (k_2S_-k_2F_)/k_2F_ ·100), k_3_ (ε_k3S_ = (k_3S_-k_3F_)/k_3F_·100) and F_e_ (ε_Fe_ = (F_e_-F_ei_)/F_ei_·100) versus k_5_ at a k_5_/k_6_ ratio of 0.14, 0.69 and of 1.24; (B) absolute errors of k_1_ (Δ_k1_ = k_1S_-k_1F_), k_2_ (Δ_k2_ = k_2S_-k_2F_), k_3_ (Δ_k3S_ = k_3S_-k_3F_) and F_e_ (Δ_Fe_ = F_e_-F_ei_) versus k_5_ at a k_5_/k_6_ ratio of 0.14, 0.69 and of 1.24.

Assuming the existence of an extra-vascular extra-cellular compartment, estimation errors in parameters k_3_ and F_e_ of the Sokoloff model would occur because tracer in that extra-vascular extra-cellular compartment would be assigned to other compartments of the Sokoloff model in order to explain the regional tracer kinetics (Fig. 7). This reallocation of activity and its effect on parameter errors was dependent on both k_5_ and k_5_/k_6_ (Fig. 7). For low values of k_5_, more of the non-substrate activity was reallocated to the metabolized compartment than to the extra-vascular substrate compartment at both values of k_5_/k_6_ studied (Fig. 7A). As k_5_ increased, the reallocation of the non-substrate activity shifted more to the extra-vascular substrate compartment than to the metabolized compartment. Relative errors in the parameters k_3_ and F_e_ were consistent with these reallocations in activity (Fig. 7B). When more activity was reallocated to the metabolized compartment, the estimated value of k_3_ necessarily increased, resulting in higher relative error in k_3S_ than in F_e_ (Fig. 7B). Likewise, when more activity was reallocated to the extra-vascular substrate compartment, F_e_ demonstrated larger error (Fig. 7B). In fact, when the relative error of F_e_ exceeded the relative error in Ki, the relative error in k_3_ became negative (Fig. 7B). In such cases, the large overestimation of F_e_ necessitated k_3_ values smaller than the real value to yield the given net uptake. The overestimation of F_e_ primarily resulted from greater relative and absolute errors in k_2_ than in k_1_ (Fig. 8).

The k_5_/k_6_ ratio did not seem to change these general patterns, but did influence peak error in k_3_, F_e_, and Ki_S_, as well as the specific k_5_ values at which these peaks occurred (Fig. 7B).

## Discussion

The main findings of this study in mechanically ventilated sheep with different types of regional acute lung injury are:

1. in the majority of studied ROIs (85.7%), the four-compartment model provided a better description of ^18^F-FDG kinetics, and reduced the overestimation of net uptake rate Ki compared to the Sokoloff model; (2) in a large fraction of severely injured (LPS+, Lav+) ROIs, the phosphorylation rate k_3_ was underestimated and the distribution volume F_e_ was overestimated with the Sokoloff model relative to the four-compartment model; (3) the Ki of the Sokoloff model resulted in a larger bias than the Ki of the Patlak method when compared to the four-compartment model; and (4) in simulations, errors in Ki of the Patlak method and Ki, F_e_, and k_3_ of the Sokoloff method were dependent on the magnitude of k_5_ and the k_5_/k_6_ ratio, with smaller errors in Patlak Ki than Sokoloff Ki over all k_5_ and k_5_/k_6_ values.

Previous studies have established that ^18^F-FDG uptake during lung inflammation in the non-tumoral lung is predominantly due to the number and degree of activation of neutrophils [4], [8], [10], [12]. Given that ^18^F-FDG is not a specific tracer for inflammation, any process leading to accumulation of ^18^F-FDG within a ROI can potentially increase the measured ^18^F-FDG uptake rate. Such processes may include diffusion of tracer into regions of alveolar flooding or edema as well as tracer uptake by other cells such as endothelial cells [31] or macrophages [32]. Those cells were thought to have a minor effect in conditions of substantial lung inflammation [4], [8], [10]. However, it remains unknown how alveolar flooding or edema influence measurements of ^18^F-FDG uptake in early stages of lung injury. Additionally, the effect of lung water on the estimation of ^18^F-FDG uptake may depend on the specific model being used as well as the severity of inflammation. Understanding the effect of lung water on the reliability of ^18^F-FDG uptake is important, as ^18^F-FDG uptake has been proposed as an early predictor of ALI [5], [33], [34].

In addition to the ^18^F-FDG net uptake rate Ki, other parameters of the ^18^F-FDG models may be affected by increased lung water. In particular the estimation of k_3_ and F_e_ or F_ei_, all components of Ki, are likely to be affected. The parameter k_3_ represents the phosphorylation rate of ^18^F-FDG, an indicator of the level of metabolic activity of neutrophils. F_e_ and F_ei_ represent the volume of distribution of ^18^F-FDG immediately available for phosphorylation, for which the number of neutrophils is a predominant factor. Since those aspects of inflammation are potentially relevant to study ALI, it is important to understand how increased lung water may affect the estimation of these parameters in addition to Ki. To address this problem, we compared estimates of the Patlak, Sokoloff and four-compartment methods in four distinct conditions. To account for the broad range of model estimates due to regional heterogeneity in lung function and metabolic activity [33], [34] those parameters were quantified in three isogravitational ROIs.

Combinations of brochoalveolar lavage (Lav− and Lav+) and continuous systemic endotoxemia (LPS− and LPS+) were used to produce varying degrees of inflammation and alveolar or interstitial edema. Bronchoalveolar lavage caused a large amount of regional alveolar flooding, as attested by the increased wet-to-dry ratio and lung density as well as the volume of saline left in the lavaged lung (400 mL [300–600]) when compared with the functional residual capacity of an adult sheep lung (∼550 mL) [35]. Lavage is also known to promote mild pulmonary neutrophilic infiltration [34], [36] potentially due to surfactant depletion and mechanical injury [34]. Endotoxemia causes significant neutrophil infiltration in the lungs [33] and a dose-dependent increase in the glucose uptake in neutrophils [37]. Also, it has been shown to produce interstitial edema but not edema in air spaces due to an injury of the capillary endothelium rather than alveolar epithelium [38]. To minimize additional lung injury by mechanical ventilation, we used pressures within accepted clinical limits.

The four-compartment model provided a better description of ^18^F-FDG kinetics than the Sokoloff model in 85.7% of the ROIs based on the AIC. The AIC provides an objective measure to identify the model with the better tradeoff between minimizing the fit error and the number of model parameters [29], and has previously been used for ^18^F-FDG model selection [12]. The fact that the four-compartment model resulted in a lower AIC than the Sokoloff model in those ROIs, in spite of the penalty included in the AIC for its two additional parameters, indicates that the improvements in the fitting of ^18^F-FDG kinetics were substantial and the additional compartment was warranted to predict those kinetics.

In the “LPS+, Lav+” condition of the experimental studies the Sokoloff model appeared to overestimate F_e_ and to underestimate k_3_ as compared to the four-compartment model. Simulations also showed that significant errors may occur in F_e_ and k_3_, even when there is minimal error in net uptake Ki, and the magnitude of those errors depends on the properties of the extra-vascular extra-cellular compartment. As demonstrated by the absolute and relative errors of k_2_, the overestimation of F_e_ ( = k_1_/(k_2_+k_3_)) was primarily due to an underestimation of the ^18^F-FDG transport from the extra-vascular precursor compartment back to the blood. The underestimation of k_3_, i.e. of the ^18^F-FDG phosphorylation, had minor effects on the errors in F_e_.

We focused our analysis on F_e_ and k_3_ since those parameters likely provide information about specific aspects of neutrophilic inflammation, i.e., F_e_ for neutrophil number and k_3_ for their degree of activation [12]. We speculate that these parameters may also be important for evaluation of lung injury in addition to the net uptake rate Ki. In fact, recent data suggests that k_3_ could be important to characterize the regional effects of mechanical ventilation during early endotoxemia [39], in line with the finding of the prognostic value of k_3_ in cancer research [40]. Moreover, estimation errors in those parameters determine the error in Ki.

The observed smaller biases in net uptake rate Δ_KiP_ estimated with the Patlak method compared to the Sokoloff model suggest that the Patlak method may be more robust than the Sokoloff model across various conditions. Our simulations confirmed in both uninjured and severely injured conditions systematically lower errors in Δ_KiP_ compared to Δ_KiS_ of the Sokoloff model over a wide range of k_5_ and k_5_/k_6_. The robustness of the Patlak method may result from the fact that it does not attempt to fit the first 15 minutes of ^18^F-FDG kinetics, so that changes in that early phase caused by the presence of lung water have no effect on the estimation of Ki. For the Sokoloff model, those changes in early kinetics may be a cause of overestimation of Ki, particularly in regions with high lung water content.

The four-compartment model includes an extra-vascular extra-cellular compartment that accounts for increased lung water in a lung region, in contrast to the Patlak and Sokoloff methods. The effect of this compartment on overall tracer exchange should be largely determined by its functional volume relative to that of the precursor compartment (F_ee_/F_ei_), which is determined by the ratio of k_5_ and k_6_. In addition, the magnitudes of k_5_ and k_6_ determine the time-scale of tracer dynamics in this compartment, i.e. whether the compartment quickly equilibrates with the precursor compartment or slowly accumulates tracer over time.

In line with those concepts, we found in our simulations that errors in parameters of the Patlak and Sokoloff methods caused by the presence of the extra-vascular extra-cellular compartment depend not only on the volume of distribution of that compartment (k_5_/k_6_), but also on the dynamic response properties of the compartment. These findings suggest that when alveolar flooding and interstitial or alveolar edema may be present, consideration of the four-compartment model is necessary to avoid significant errors in parameter estimates of both the Patlak and Sokoloff methods. Although the additional parameters of the four-compartment model could increase the uncertainty of the estimated model parameters, the comparison of the AIC values clearly showed that the higher model order is justified. The large number of data points in the imaged ^18^F-FDG kinetics (n = 40), as well as the low noise levels in large, isogravitational ROIs, likely contribute to the reliability of parameter estimates in the four-compartment model, as well as in the Patlak and Sokoloff methods. When signal-to-noise ratios in tracer kinetics are lower, such as in small ROIs, the simpler models may have advantages over the four-compartment model in terms of robust parameter estimation. In general, since the four-compartment model provided better fits even in some uninjured ROIs, the selection of either the Sokoloff or four-compartment model for a given tracer kinetics curve should ideally be made using a statistical criterion such as the AIC.

Absolute and relative errors of estimated parameters are both highly relevant characteristics of error properties (Table 3). The high absolute error of Δ_KiS_ for “LPS+, Lav+” compared to “LPS−, Lav−” simulations may, for example, affect statistical tests among groups or conditions with different Ki_S_ and lead to false conclusions. Interestingly, relative errors ε_KiS_ show the opposite relationship between the two conditions. This illustrates that in regions of low ^18^F-FDG uptake, tracer accumulation in the extra-vascular extra-cellular compartment has a proportionally greater effect on the error of Ki_S_. Also, neither absolute nor relative errors of the estimated parameters are independent of the parameter value.

Our findings emphasize the need to account for the presence of lung edema and alveolar flooding in the quantification of ^18^F-FDG kinetics as a marker of lung inflammation. For the Sokoloff model, ^18^F-FDG diffusion into the extra-vascular extra-cellular space leads to estimation errors in the ^18^F-FDG net uptake rate and in parameters describing important components of pulmonary inflammation: F_e_, which may be useful to characterize the number of inflammatory cells; and k_3_, assumed to reflect the activation degree of those cells. This might be important for studies focusing on a detailed description of inflammatory processes, or for the assessment of new anti-inflammatory therapies. Moreover, interpretation of ^18^F-FDG data in patients with ALI/ARDS [3], in whom lung edema and flooding are common, could be advanced by using the lung-specific four-compartment model.

In summary, the findings of our experimental and theoretical studies suggest that increased lung water affects parameter estimates of the Patlak and Sokoloff models of ^18^F-FDG kinetics. The lung-specific four-compartment model, which includes an extra-vascular extra-cellular compartment to account for effect of lung water on ^18^F-FDG kinetics, provides better description of ^18^F-FDG kinetics compared to the Sokoloff model, both in terms of the net ^18^F-FDG uptake rate and of its component transfer rates. The Patlak method resulted in relatively small errors in net uptake rate, but does not allow for assessment of more specific parameters of ^18^F-FDG kinetics. The advantages of the four-compartment model are relevant for investigation of regional inflammation during acute lung injury with positron emission tomography.

## Supporting Information

Figure S1 **Relative Errors in Net Uptake Rates Estimated by the Patlak and Sokoloff Methods.** Relative errors in Ki of the Sokoloff (ε_KiS_ = Ki_S_-Ki_F_/Ki_F_·100 [%]) and the Patlak method (ε_KiP_ = Ki_P_-Ki_F_/Ki_F_·100 [%]) compared to the four-compartment model (contour lines) as function of k_5_/k_6_ and k_5_ in simulations of a healthy lung (LPS−, Lav−) and of a lung exposed to systemic endotoxin and bronchoalveolar lavage (LPS+, Lav+).
(TIF)
