# Supplementary figures and images for: Modeling 18F-FDG Kinetics during Acute Lung Injury: Experimental Data and Estimation Errors
Source: PLoS One. 2012 Oct 31;7(10):e47588. doi: 10.1371/journal.pone.0047588 (PMC3485257; doi:10.1371/journal.pone.0047588)

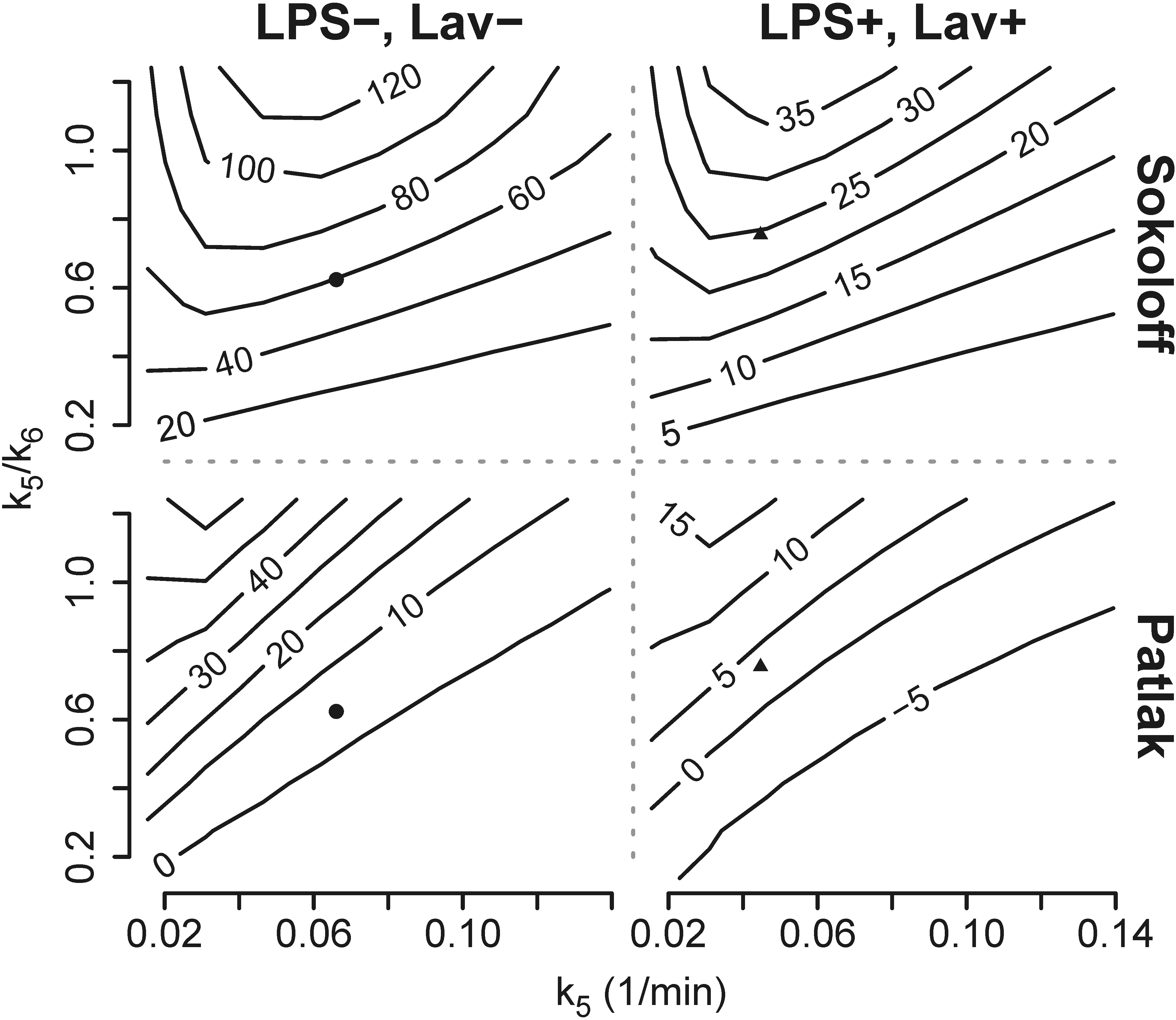

Supplement: Figure S1 — Relative Errors in Net Uptake Rates Estimated by the Patlak and Sokoloff Methods. Relative errors in Ki of the Sokoloff (εKiS = KiS-KiF/KiF·100 [%]) and the Patlak method (εKiP = KiP-KiF/KiF·100 [%]) compared to the four-compartment model (contour lines) as function of k5/k6 and k5 in simulations of a healthy lung (LPS−, Lav−) and of a lung exposed to systemic endotoxin and bronchoalveolar lavage (LPS+, Lav+). (TIF) [file pone.0047588.s001.tif]
